# The biological sense of cancer: a hypothesis

**DOI:** 10.1186/1742-4682-3-43

**Published:** 2006-12-15

**Authors:** Raúl A Ruggiero, Oscar D Bustuoabad

**Affiliations:** 1 División Medicina Experimental, Instituto de Investigaciones Hematológicas, Academia Nacional de Medicina de Buenos Aires, Pacheco de Melo 3081, 1425 Buenos Aires, Argentina

## Abstract

**Background:**

Most theories about cancer proposed during the last century share a common denominator: cancer is believed to be a *biological nonsense *for the organism in which it originates, since cancer cells are believed to be ones evading the rules that control normal cell proliferation and differentiation. In this essay, we have challenged this interpretation on the basis that, throughout the animal kingdom, cancer seems to arise only in injured organs and tissues that display lost or diminished regenerative ability.

**Hypothesis:**

According to our hypothesis, a tumor cell would be the only one able to respond to the demand to proliferate in the organ of origin. It would be surrounded by "normal" aged cells that cannot respond to that signal. According to this interpretation, cancer would have a profound *biological sense*: it would be the ultimate way to attempt to restore organ functions and structures that have been lost or altered by aging or noxious environmental agents. In this way, the features commonly associated with tumor cells could be reinterpreted as progressively acquired adaptations for responding to a permanent regenerative signal in the context of tissue injury. Analogously, several embryo developmental stages could be dependent on cellular damage and death, which together disrupt the field topography. However, unlike normal structures, cancer would have *no physiological value*, because the usually poor or non-functional nature of its cells would make their reparative task unattainable.

**Conclusion:**

The hypothesis advanced in this essay might have significant practical implications. All conventional therapies against cancer attempt to kill all cancer cells. However, according to our hypothesis, the problem might not be solved even if all the tumor cells were eradicated. In effect, if the organ failure remained, new tumor cells would emerge and the tumor would reinitiate its progressive growth in response to the permanent regenerative signal of the non-restored organ. Therefore, efficient anti-cancer therapy should combine an attack against the tumor cells themselves with the correction of the organ failure, which, according to this hypothesis, is fundamental to the origin of the cancer.

## Background

Cancers as well as benign neoplasias are very old diseases, which have afflicted animals since long before man appeared on earth [1,2] and human beings since prehistoric times [1,3]. Written records concerning cancer can be traced to ancient Egypt [4]. However, there is consensus that only during the past 100 years has a truly scientific approach to malignant diseases emerged as a result of the mounting and concerted efforts of clinical physicians, experimentalists and theoretical scientists. Since the late 1970, different alterations in cellular genes as well as in several intracellular transducing signaling pathways have been identified in cancer cells, and on this basis a unified genetic theory of carcinogenesis has been advanced [5-8].

This theory states that cancer starts and ends with the malignant cell, in which genetic changes lead to constitutive activation of some genes (oncogenes) and/or inactivation of others (anti-oncogenes or tumor suppressor genes). allowing that cell to evade – in all or in some microenvironments – the mechanisms controlling cell proliferation. These genetic changes would define the molecular and cellular attributes of the cancer cell, which, in turn, should be the target of specific therapies against cancer. This theory has the enormous merit of unifying, through an immediate common cause, the numerous different mediate causes of cancer such as chemicals, radiation, viruses, etc. However, it has some theoretical difficulties, which have been addressed [9-11] by authors who have also emphasized that cancer remains a major cause of morbidity and mortality, despite the explosive development of our knowledge about the molecular mechanisms associated with the control of cell cycle and survival [12]. Of course, these theoretical difficulties and the persistent failure in treating cancer do not necessarily imply that the unified genetic theory of carcinogenesis is incorrect. However, they encourage us to explore other possible theoretical approaches.

In this paper, on the basis of ideas advanced by Prehn, Zajicek, Bissell, Duesberg, Sonnenschein and Soto [9,13-16] among others, we propose a hypothesis of cancer that does not consider it an autonomous entity disobeying the mechanisms controlling cell proliferation, but one dependent on a reparative signal originating in the particular environment of an injured tissue with diminished or exhausted reparative ability. Hopefully, this hypothesis might help to reconcile some apparently contradictory approaches entailed in the unified genetic and some alternative theories of carcinogenesis, improving our understanding of the relationship among aging, regeneration and cancer.

## Postulates

This hypothesis is based on three postulates:

**1) **Throughout the animal kingdom, cancer is rarely – if ever – produced in body regions displaying *strong *regenerative ability, "strong" meaning the ability to regenerate complex structures such as a whole limb. These regions can encompass the whole body, as in sponges, cnidarians, echinoderms, nematodes, sipunculides [17-20], etc. or parts of the body, as in the upper body regions of Planaria, phylum Platyhelminthes [21]; hind limbs of urodele amphibians [13,22]; etc. Conversely, cancer is relatively frequent in animals that display *weak *regenerative ability throughout their bodies, such as vertebrates others than urodele amphibians, arachnids, insects [13,19,23-26], etc., "weak" meaning the ability to repair or regenerate relatively simple structures only, as in compensatory hyperplasia of the liver, skin regeneration, etc. A similar relatively high frequency of tumors has been observed in the body regions of urodele amphibians that cannot regenerate [27,28].

**2) **In animals in which cancer is relatively frequent, cancer incidence rises exponentially with age [29]. In addition, when cancer develops in young animals, it is usually associated with injured organs and tissues such as cirrhotic liver, gastric tissues exhibiting chronic atrophic gastritis, radiation-damaged skin, colon displaying ulcerative colitis, breasts of nulliparous women, non-secreting prostate alveoli, etc., which may have exhausted or diminished their regenerative abilities [13,30,31].

**3) **In animals displaying a strong regenerative ability, reparative or/and regenerative mechanisms remain fairly efficient throughout life [32]. On the other hand, in animals displaying a weak regenerative ability, reparative or/and regenerative mechanisms are efficient mainly during youth; as these animals age, cellular loss increases and those mechanisms wane progressively [33].

## Corollaries

**1) Throughout the animal kingdom, cancer is rarely – if ever – induced in organs (or tissues) displaying an efficient reparative or regenerative mechanism, "efficient" meaning the ability of organs and tissues to regenerate themselves numerically and functionally**. In effect, when these mechanisms remain fairly efficient throughout life – even under the action of putative noxious agents – as they do in animals displaying strong regenerative ability, cancer never (or almost never) occurs. When they remain efficient only during youth – and even during youth, some noxious agents can deplete them – as they do in animals displaying weak regenerative ability, cancer occurs mainly in aging individuals and also in injured organs from young individuals that may have exhausted their regenerative ability because of the action of those noxious agents.

**2) **Homeostasis in organs or tissues with mitotic potential would be maintained by regulatory fields, "regulatory field" meaning the existence of inhibitory and stimulatory signals for cell proliferation and differentiation within the space of an organ or tissue. Both types of signal, regardless of their molecular nature, would not be symmetric. In effect, when a reparative or regenerative mechanism is efficient, all cellular loss is compensated by cellular division until the organ attains its original size and function, after which all new mitoses are inhibited. **This inhibitory signal, associated with the "right" number of normal functional cells located in the "right" place, must be obeyed not only by the normal cells of the organ but also by all putative anomalous cells that could have emerged within the organ by chance, injury or other cause. **In effect, if these anomalous cells could disobey the inhibitory signal and grow autonomously, cancer could develop rather easily in an organ exhibiting an efficient reparative or regenerative mechanism, contradicting *corollary 1*. In contrast, the mere existence of an organ displaying an inefficient reparative mechanism means that some or all of their cells could occasionally be non-responsive to the stimulatory signal associated with (or produced by) the "less than right" number of functional cells of that organ. The concept of the "right" number of cells in the "right" place can be elucidated by the following example: when a liver is intact, no proliferation of hepatocytes occurs; when it is partially excised and regenerative ability is normal, proliferation occurs until the liver attains its original size and function. The number of hepatocytes in the intact liver would be the "right" number of functional cells, which would induce or produce an inhibitory signal(s) for the hepatocytes. Proliferation of hepatocytes after partial hepatectomy would not be prevented by ectopic implantation of liver cells, meaning that these ectopic cells would not be in the "right" place for sending inhibitory signals to prevent hepatocyte proliferation in the remnant liver.

## Origin of tumor cells

What, according to this hypothesis, is the putative origin of cancer?

We have said that cancer would not be induced in organs (or tissues) exhibiting an efficient regenerative mechanism. However, when an organism becomes aged and its regenerative ability is progressively lost, any injury causing loss of cells or cellular function cannot be compensated by cellular division. In consequence, the original size and function of the organ cannot be restored.

We suggest that this situation induces a "**crisis**", which, through putative danger signals resulting from retardation of tissue repair, acceleration of cell loss and functional compromise, might create an environment capable of promoting some degree of variability in the remaining live but arrested cells of the injured organ. The outcome of this situation would be the emergence of some genetically and/or epigenetically modified cell variants. Most of these would still lack the ability to divide in response to the organ demand, but sooner or later a variant bearing that mitotic ability would emerge by chance. This new variant would begin to divide; and if it were poorly functional or non-functional, the organ would be numerically but not functionally restored. In consequence, it would not score the regeneration as effective and it would continue to send mitotic signals to restore the lost or diminished organ function. As a result, the new variant would grow over and over and the outcome would be a tumor. On the other hand, if the emergent new variant were functionally active, normal function might be restored and this "restored" organ might, in most cases, mimic the negative regulatory field associated with the intact organ, after which further mitosis would be halted. In a few cases, however, the new variant – even if functional – might be unable to mimic that negative regulatory field (for example, because of aberrant cellular features not directly related to function) and in such cases a tumor would also be produced. In the case of poorly functional or non-functional variants, the tumor would be poorly functional or non-functional, as most tumors are. On the other hand, in the special cases of functional variants producing tumors, they would be functioning ones, such as some adenomas or some papillary and follicular carcinomas of the thyroid.

Many authors have highlighted the critical importance of injury in the development of cancer [31,34-37], and the idea that cancer actually behaves as a wound healing process has been suggested by Dvorak [38]. Others have challenged this interpretation [39,40], but a critical examination of their data reveals that they scored only massive necrosis and overt degenerative changes as "injury", dismissing less evident injuries such as lost or diminished function of the whole organ or part of the organ, apoptosis, cellular senescence, etc. These are as relevant as massive or overt injury for this hypothesis, because both demand a regenerative response.

Cellular heterogeneity, and a genomic instability phase during stages of high-grade dysplasia prior to the acquisition of a frankly malignant phenotype, are two well-documented (though so far unexplained) phenomena [33,41]. Similarly well-documented are the picture of a tumor arising in a tissue surrounded by "normal" arrested cells, and the existence of factors involved in organ and tissue regeneration that enhance or are necessary for tumor growth [15,36,42]. Moreover, under certain conditions, the immune response might play a role in tissue regeneration, and in that case it would stimulate rather than inhibit tumor growth [43,44].

In summary, according to this hypothesis, cancer would originate on the basis of three conditions:

a) An injury of the affected organ (or tissue), "injury" meaning not only partial removal of the organ, massive necrosis or extensive degenerative change but also less evident deleterious effects such as lost or diminished function of the whole or a part of the organ, apoptosis, cellular senescence, etc.

b) The impossibility of restoring the injury to that organ, and the consequent existence of a permanent reparative signal to the remaining live cells.

c) The existence or emergence of atypical cells able to respond to the mitotic reparative signal of the injured organ but unable to mimic the negative regulatory field associated with the intact organ.

Our hypothesis about the origin of cancer seems to work regardless of which hypothesis we adopt for the control of the cell proliferation. In effect, if we adopt the stimulatory or positive hypothesis [45], the regenerative signals will be represented by different kinds of growth factors depending on the tissue or organ involved. In the same way, the diminished or lost expression of at least one of the numerous molecular steps in the growth factor signaling pathway in normal aged cells – and, conversely, the existence of a responsive pathway in cancer cells – might explain why the latter can proliferate in an organ where normal aged cells cannot. On the other hand, if we adopt the inhibitory or negative hypothesis [45], the regenerative signals will be represented by the absence of some kinds of inhibitory factors (chalones, TGF-β among others). In the same way, the constitutive expression of at least one step in the inhibitory signaling pathway in normal aged cells – and, conversely, the absence of such constitutive expression in tumor cells – might explain why tumor cells can proliferate while normal aged cells cannot.

A plausible objection may be raised about the origin of cancer postulated by this hypothesis. If cancers originate in injured organs or tissues that have exhausted or diminished regenerative capacities, they should be much more frequent in organs or tissues that display poor or null regenerative ability from birth. An obvious example is neuronal tissue in the human brain; however, this tissue actually exhibits fewer tumors than other organs and tissues such as colon, breast, lung and skin [12,46]. The answer to this objection might be as follows: as stated in *corollary 2*, "regulatory fields" seem to be necessary to control the proliferation of cells with mitotic potential, which are found in almost all body organs and tissues. However, the theory does not require that "regulatory fields" control the proliferation of postmitotic cells such as brain neurons, because they would not proliferate on their own, as shown by their inability to re-enter the cell cycle even upon stimulation [47]. Therefore, while the neuronal tissue of the brain remains intact, no extracellular inhibitory signals seem to be necessary to keep its cells arrested. On the other hand, when that tissue is injured, probably no stimulatory signals will be generated. In consequence, according to the hypothesis, no primary condition exists for tumor initiation.

## Properties of tumor growth

Since tumor growth does not restore the negative regulatory field associated with the intact organ, the "crisis" would persist and, as a consequence, new variants would be forced to emerge continuously by chance in the "normal" resting tissue as well as within the growing tumor. In fact, new cellular variants have been found in the "normal" tissue surrounding a tumor [48,49]. In the same way, new variants continuously emerging in the tumor itself could account for the cellular heterogeneity typically observed in both experimental and clinical tumors [50].

In addition, since the speed of regeneration of a partially removed organ or tissue is greatest at the outset of the process, when the lack of function is maximal [51], our hypothesis would predict that the more undifferentiated and non-functional the tumor cell, the faster its growth, because for all practical purposes, "regeneration" by non-functional tumor cells would always simulate the outset of the normal regeneration process. The faster growth of more undifferentiated tumors compared with more differentiated ones is a common but not yet satisfactorily explained phenomenon in tumor biology [46,52].

## The nature of the tumor cell

The most intriguing consequence of this hypothesis concerns the nature of the tumor cell itself. During the past century, many quite different theories and hypotheses about cancer have been proposed (reviewed in [45,46,51]). Despite their wide differences, most of these accounts agree that a frank or true tumor cell is autonomous, meaning that it is not subject to the rules and regulations that control normal cell proliferation and survival. The concept of autonomy was originally enunciated in a biological sense (classical definition of Ewing [53]), but the main goal of experimental oncology has been "to understand it in the molecular sense", that is "to elucidate the molecular definition of the cancer cell regardless of its environment" [46].

With the help of new molecular technologies, several intracellular transducing pathways have been elucidated in the last 25 years and progress in dissecting these pathways "has begun to lay out a circuitry that will likely mimic electronic integrated circuits in complexity and finesse, where transistors are replaced by proteins (e.g. kinases and phosphatases) and the electrons by phosphates and lipids, among others" [6]. Some of these pathways transmit stimulatory growth signals from the extracellular medium to the nucleus, such as the mitogen-activated protein kinase (MAP-kinase) cascade. Others transmit inhibitory signals (most of them funneled through the retinoblastoma protein, pRB, and its two relatives, p107 and p130), death signals (such as that initiated by Fas L), survival signals (such as that initiated by IGF-1), etc. [6,54]. In this context, the constitutive expression of any step(s) in the stimulatory and/or survival signaling pathways (most of them related to the expression of known "protooncogenes"), or the constitutive blockade of any step(s) in the inhibitory and/or death signaling pathways (most of them related to the expression of some known "antioncogenes"), or a combination of both, would confer the capacity for autonomous growth on the cell.

Some authors have claimed that this autonomy is not absolute but relative, meaning that the expression of some oncogenes or the silencing of some antioncogenes may generate cancer in some but not all environments. This contention was originally suggested by the classical experiments of Brinster and Mintz and Illmense, demonstrating that the malignant potential of teratocarcinoma cells could be constrained if they were injected into the blastocyst; the resulting mice contained tumor-free tissues derived from the teratocarcinoma cells [15]. Further evidence is available to support this claim. For example, infection of adult chickens with Rous Sarcoma Virus (RSV) leads to malignant transformation associated with the expression of the oncogene v-src; however, infection of chick embryos *in ovo *with RSV does not lead to malignant transformation, even though v-src is both expressed and active [15]. In the same way, expression of v-myc and c-myc is typical of some tumors, but myc is also expressed in echinoderms, which never develop tumors [17]. In any case, irrespective of whether a tumor cell is considered absolutely or relatively autonomous, there is a consensus that it has molecular anomalies that allow it to escape – in all or in some environments – from the regulatory mechanisms that inhibit normal cell proliferation in those environments.

However, if the hypothesis advanced in this paper were true, a tumor cell would not be one ignoring the mechanisms that control normal cell proliferation. In fact, in the injured organ where tumor originates, the tumor cell would be the only one able to respond to the organ demand to proliferate, surrounded by "normal" aged cells that cannot respond to that signal. *In this way, any attempt to find the molecular definition of the cancer cell, meaning the molecular anomalies that allow the tumor cell to escape from the inhibitory signals of normal cell proliferation, might be an attempt to find something that does not exist*. Of course, there are many reported genetic and even heritable epigenetic changes in different tumors [55,56], but these changes might not be the origin of cancer. Instead, they could be reinterpreted as adaptations of cancer cells that enable them to respond to the demand of the aged organ to proliferate in response to injury. Claims that several putatively oncogenic mutations could be the result rather than the cause of cancer are available in the literature [9-11,57].

According to our hypothesis, any non-functional (and a few aberrant functional) but mitotically active variant present in an injured "aged" organ – with exhausted or diminished regenerative capacity – could behave as a tumor cell. But the same cell put into a "young" organ with an intact regenerative capacity would behave as a normal cell. Moreover, in very special situations, even absolutely normal functional cells could behave as tumor cells. For example, when an inert foreign body (such as a glass cylinder) is subcutaneously implanted in a mouse, tissue homeostasis is disrupted and, in consequence, a regenerative signal must be produced. If the tissue is "young", absolutely normal cells will proliferate to repair it, but the presence of the foreign body would not allow the repair to be effected. Therefore, the regenerative signal would continue (presumably because although there are sufficient normal functional cells to heal the injury, they are in the "wrong" place), and a tumor-like proliferation of exclusively normal cells would result. The "crisis" generated by the unresolved disruption of homeostasis would persist, and eventually new non-functional variants would emerge, better adapted to respond to the regenerative stimulus; these would be the origin of the late sarcomas observed in such cases [58,59]. The existence of a tumor-like proliferation of normal mesenchymal cells, relatively early after foreign body implantation, is a well-documented observation [58].

Our suggestion that a tumor cell is not autonomous but dependent on a reparative or regenerative signal originating in an "aged" organ or tissue seems heretical, because it contradicts the classical definition of Ewing ("A neoplasm is an autonomous, or relatively autonomous, growth of tissue"), which has guided cancer research for the last 60 or more years [53]. However, closer examination of Ewing's proposition reveals that it is a postulate rather than a true definition. First, pathologists do not use it as an operational tool to diagnose the presence of a tumor; in fact, "the means to diagnose cancer have not changed that much since"... the 19^th ^century, "when pathologists began describing the histological pattern of tumors using the light microscope" [45]. Second, if nobody knows exactly what the mechanisms control normal cell proliferation [45], how can anyone be absolutely sure that cancer cells are disobeying those mechanisms? Some years ago, Dr Joseph Aub suggested that the "ugly word autonomy" be dropped, because while one can prove dependency, one is never certain of autonomy [60].

## The riddle of the blue whale and the mouse

The unified genetic as well as some (but not all) alternative theories of carcinogenesis share the idea that the malignant cell is the physiological and anatomical unit of cancer disease. Implicit in this contention is the assumption that the probability of origin of an aberrant, neoplastic cell lineage is the same per unit of cell population, regardless of species or cell type concerned.

However, this assumption evokes one of the most intriguing riddles in cancer research, which remains unsolved. This riddle, stated by Dawe [20] some years ago, asks: "Why don't extremely large animals develop neoplasms with a much higher incidence than very small ones since the cell population at risk is greater by several orders of magnitude?" As an extreme example, let us consider the blue whale and the mouse. "If one takes the weight of the mouse as 30 g and that of the blue whale as 100 tons, the whale is equivalent to 3,030,303 mice. Then, if one accounts for differences of lifespan (65 years for the blue whale, 3 years for the mouse), the ratio of weight-year units per whale to weight-year units per mouse is about 66,670,000" [20]. We should therefore expect the blue whale to develop neoplasias about 3 × 10^6 ^and 6.6 × 10^7 ^times more often than the mouse per unit time and per lifespan, respectively. Since about 40% of wild mice kept under laboratory observation develop spontaneous neoplasias during their lives [61], we should expect each blue whale to develop about 2.6 × 10^7 ^neoplasms per lifespan. It is clear that these expectations do not match reality: the incidence of neoplasia in whales, as in most mammals, is roughly similar to that in mice. Therefore, the incidence of neoplasia is not a simple function of protoplasm mass at risk per unit time. In fact, the greater the body size of the animal, the greater seems to be its resistance to oncogenesis on a unit weight per unit time basis.

Some *ad hoc *hypotheses have been invoked to account for this fact on the assumption that *the individual cell in an organ or tissue is the unit at risk of carcinogenesis*. For example, the animal fat depots might sequester fat-soluble carcinogens with an efficiency proportional to animal's size and thereby proportionately diminish the exposure of other tissues. Another possibility is that the efficiency of defenses against neoplasia, such as mechanisms of DNA repair, cellular resistance to metabolism and mutagenic activation of putative carcinogens, immunological surveillance, etc., could be proportional to animal size. While these invoked mechanisms remain largely undemonstrated as general rules [62-64], the hypothesis of cancer that we present in this paper could offer a relatively easy solution of the riddle (although not necessarily excluding other interpretations [62,65], which in fact might complement ours) by assuming that *the true basic unit at risk of carcinogenesis is the tissue or organ as a whole rather than the individual cell*. In effect, according to the hypothesis, cancer originates in organs or tissues that have exhausted or diminished their regenerative capacities, and this would occur when all or a critical proportion of their cells have partially or wholly lost that capacity. In such a case, if an organ were x times larger than another one, the probability that its regenerative capacity is critically diminished would be x times lower, because an x times greater number of cells would have to be affected to depress that capacity. This lower probability would balance the proportionally higher number of their cells that could be transformed. As a result, if the unit at risk is, for example, one liver rather than 10^9 ^(mouse) as opposed to 3 × 10^15 ^(blue whale) liver cells, then the whale will be at no greater risk of developing liver cancer than the mouse, or any other animal with an equally efficient defense mechanism against neoplasia. The idea that cancer is an organ or tissue disease rather than a cellular one has been advocated especially by the group of Sonnenschein and Soto [45].

## Tumor progression. Invasion and metastases

Sooner or later, tumor growth will be restrained by the rather rigid architecture of the organ or tissue in which the tumor originated (first tissue). However, the persistent "crisis" will force the emergence of new variants with the ability to disrupt that architecture, so growth can be re-initiated. When these new variants reach the basal membrane, they would eventually be able to disrupt it, allowing the tumor cells to invade another tissue (second tissue). The claim that cancer cells can produce enzymes that destroy the matrix barriers surrounding the tumor, permitting invasion into surrounding tissues, has significant experimental support [66,67].

Assuming that the second tissue is not injured and that its regenerative capacity is intact, the invading tumor cells would face an inhibitory signal from the second tissue which – according to *corollary 2 *– they could not disobey. At that point, the tumor cells might remain arrested indefinitely. Alternatively, the arrested tumor cells might produce – directly, by releasing inhibitory factors, or indirectly, by attracting inflammatory cells that in turn release inhibitory factors – a lowering of the regenerative capacity of the second tissue. If an injury were incurred in the second tissue, simultaneously or subsequently – most probably associated with the pre-acquired ability of the tumor cells to disrupt the architecture of the first tissue – a stimulatory signal would appear, aimed at repairing the injured tissue. Since the regenerative capacity of the tissue would thereby become exhausted or diminished, the tumor cells would have a selective advantage over normal cells to proliferate. Examples of this selective advantage have been documented [68,69].

However, the tumor cells did not originate in the second tissue, and since repair or regeneration processes in different tissues are generally independent of each other [45], stimulatory signals from one tissue would not usually induce the proliferation of cells from another. Why, then, could the growth of tumor cells from the first tissue actually be stimulated by the stimulatory signal of the second? We suggest that the less the tumor resembles the primary tissue (presumably the more undifferentiated it is), the more likely it would be to respond to the stimulatory signal of the second tissue and thus to grow in it. The same procedure could also explain why tumor cells can grow in distant organs (metastases), assuming that they can reach those organs.

On the other hand, more differentiated tumor cells from the first tissue could hardly grow in the second tissue unless the stimulatory signal from the first had reached the orbit of the second. In that case, the tumor cells would grow in the injured second tissue under the guidance of the stimulatory signal from the first. This particular case can be illustrated by the behavior of so-called hormonally-conditioned tumors growing in secondary tissues or organs [60,70].

## Tumor dormancy

Tumors can occasionally remain dormant for several years, even decades; but suddenly, often in association with surgical stress or another injury, they can awake and resume progressive growth [46,71]. Some hypotheses have been advocated to explain this phenomenon [72] but its nature remains obscure.

According to the hypothesis presented in this paper, cancers originate in injured organs or tissues with exhausted or diminished regenerative capacities. However, if this exhausted or diminished capacity could sometimes be recovered, normal cells could reassume their mitotic potential and divide in response to the regenerative signal. Of course, tumor cells would also divide in response to that signal, but as the organ attained its "right size and function" – as a result of the growth of normal functionally active cells – all new mitosis would be stopped, including that in tumor cells, according to *corollary 2*. That could be the mechanism underlying the induction of a dormant tumor. The hypothesis could also offer a plausible explanation for the awakening of the dormant tumor. In effect, after years or decades of dormancy, the organ could become aged, and therefore its regenerative ability could decrease irreversibly. In that situation, any injury would induce a reparative signal to which only the hitherto "dormant tumor cells" could respond. They would thus resume their progressive growth.

Our hypothesis could operate not only for primary tumors but also for dormant metastases, the main clinical problem. In effect, as stated in the preceding section ("Tumor progression. Invasion and metastases"), when tumor cells invade a second intact tissue, they would face an inhibitory signal that they could not disobey. At that point, if these invading tumor cells were not able by themselves to injure and deplete the regenerative capacity of that second tissue, they might remain arrested indefinitely, behaving as dormant metastases. Dormant metastases may awaken as a dormant primary does, even years or decades after the tumor cells were seeded in the second tissue, when this tissue becomes aged and loses its regenerative ability.

The induction of tumor dormancy in secondary tumor implants in the presence of a primary growing tumor (concomitant resistance phenomenon [73-75]) might also be interpreted according to this hypothesis, by assuming that the local regenerative signal(s) promoting tumor growth, generated at the site of secondary tumor implantation, could be counteracted by a diffusible inhibitory factor(s) produced or induced by the large primary tumor [76].

## Transplantability of tumors

The hypothesis advanced in this paper postulates that a tumor cell is never autonomous even in the case of invasive and metastatic tumors. In effect, the mere existence of heritable changes (genetic and/or epigenetic) that endow a cell with the ability to evade the rules controlling normal cell proliferation would mean that these changes could appear by chance in a normal cell within an organ with intact regenerative capacity. But if it were possible, cancer could develop rather easily in that organ, contradicting *corollary 1*.

In this section, we consider an apparently fatal objection to the hypothesis, which is one of the milestones in the development of conventional ideas about cancer: the transplantability of experimental tumors. In 1877, Novinsky successfully transplanted tumors from adult to young dogs for the first time. These experiments were reproduced in 1888 by Moreau and later by Loeb and Jensen, using rat and murine tumors [51]. These pioneering experiments, which became universal laboratory practice for more than a century, demonstrated that only a small fragment of a tumor or a relatively small number of tumor cells dispersed in a physiological saline will suffice to transplant that tumor from a donor to a recipient host. This implies that the growth of a tumor does not need to be supported by any tissue, organ or organismic pathological condition, but only by the nature of the tumor cells themselves. In other words, tumor cells are autonomous, and this claim means that our hypothesis would be false. However, the whole of this apparently fatal objection pivots on the ambiguity of the word "autonomy".

We can accept that tumor cells are deemed "autonomous" if their inoculation into an appropriate recipient host is enough to induce new tumor growth (the first meaning of autonomy). But this does not contradict our hypothesis, because the new tumor growth need not be accomplished by evading the rules controlling normal cell proliferation in the recipient host (the second meaning of autonomy). That is, we can accept that tumor cells are autonomous in the first sense, but not in the second sense. According to our hypothesis, the mechanisms involved in tumor transplantation would not differ markedly from those used by a tumor to invade adjacent or distant organs or tissues within its primary host. In neither case would the tumor cells be autonomous in the second sense of "autonomy", because they would have to injure the recipient organ or tissue and to eliminate or reduce its regenerative capacity as a prerequisite for regenerative signals produced by the injured organ or tissue to promote tumor growth.

Our contention concerning the mechanisms underlying tumor transplantation have significant experimental support:

a) Benign tumors, which are not invasive and commonly produce little damage to host tissues, seldom – if ever – grow when transplanted into another host [77].

b) In chickens, tumors induced by Rous sarcoma virus (RSV) typically form at the viral injection site but not at distant sites; the wound associated with the injection seems to be required for local tumor growth, because additional tumors can be induced at distant sites simply by wounding the infected birds [15].

c) The liver of a young rat, but not of an aged rat in which regenerative capacity is diminished or lost, can normalize the morphology and growth capacity of transplanted hepatocarcinoma cells. The most successful normalization occurred when cells were transplanted into the spleen and filtered as solitary cells into the liver without disrupting normal liver architecture. On the other hand, when this architecture was disrupted by transplanting a greater number of malignant cells directly into the liver, normalization was less likely to occur [78].

d) Upon transplantation, tumors usually grow in anatomically correct (orthotopic) organs better than in heterotopic ones [79]. This observation can be interpreted by assuming that an invasive and transplantable tumor, even if quite different from the organ of origin, tends to be more similar to that organ than to others; in consequence, it would respond to a regenerative signal from the former better than to one from the latter, resulting in faster tumor growth.

## Carcinogenesis in vitro

Carcinogenesis *in vitro *can also be considered an objection to our hypothesis. In effect, when "transformed cells" are produced in culture – spontaneously or induced by a given carcinogen – they are assumed to be endowed with the ability to evade normal inhibitory signals when implanted into the organism. If this were true, it would be contradictory to *corollary 2*, because according to that corollary no body cell can evade such signals.

However, this conclusion is not unavoidable. It could alternatively be proposed that so-called carcinogenesis *in vitro *produces cells with particular features that enable them to disrupt homeostasis in the organ or tissue into which they are eventually implanted. This situation would initiate regenerative signals, which could be detected and utilized by the *in vitro" *transformed" cells, promoting growth in a setting in which normal cells would have been prevented from growing. That is, the putative objection of "carcinogenesis *in vitro*" could be reducible to the objection of "transplantability of tumors", which we addressed in the preceding section.

## Carcinogens

In this section we will consider another apparently fatal objection to the hypothesis presented in this paper: the existence of carcinogens. As Miller and Miller proposed [46]: "a *carcinogen *is an agent whose administration to previously untreated animals leads to a statistically significantly increased incidence of malignant neoplasms as compared with that in appropriate control animals". The most prevalent interpretation of this definition, mainly based on the putative mode of action of chemicals, radiation and oncogenic viruses, suggests that most carcinogens exert their critical effects by inducing genetic changes that endow the affected cells with the ability to grow independently of the mechanisms controlling normal cell proliferation. If this were the case, a cancer cell could emerge in the middle of an otherwise normal organ or tissue, directly contradicting *corollary 1*.

However, closer examination of the available data suggests that this prevalent view is not as straightforward as is usually thought. In effect, cancer development with chemicals, radiation, DNA viruses and retroviruses in humans and animals that lack oncogenes is a very prolonged process, often lasting one third to two thirds of the life span of the organism. This long period of development is associated with many adaptive cellular proliferative responses that may show a slow evolution to cancer [35]. For example, after treatment of rats with many different types of chemical hepatocarcinogens, rapid inhibition of cell proliferation and cellular death was observed in the liver. This early effect was followed by the appearance of clones of resistant hepatocytes, which proliferated vigorously in response to a proliferative stimulus in the hostile environment created by the carcinogen, in which the vast majority of hepatocytes, the non-resistant ones, were inhibited or dead. The resistant hepatocyte nodules have physiological value; but later, as the carcinogen-mediated injury persists, they can evolve into fully transformed cells [35,36]. Similarly, in Africa and Asia, infection with the hepatitis B DNA virus early in life is associated with the appearance of hepatocellular carcinomas 25 or 30 years later. Prevention of this disease has been achieved by a vaccine against the virus, thus preventing hepatitis and the resulting damage to the liver. This damage, caused by the cytolysis of virally-infected hepatocytes and the aberrant compensatory proliferation of the surviving hepatocytes, seems to be essential for the development of liver tumors since it is the common denominator of both virally- and non-virally-associated hepatocellular carcinomas [45,80].

On the other hand, carcinogenesis by retroviruses that carry oncogenes or v-onc genes, such as Abelson murine leukemia virus (Ab-MLV), Rous sarcoma virus (RSV), Avian erythroblastosis virus (AEV) etc., offers at first glance a very different picture, because of their ability to induce tumors rapidly and to transform cells *in vitro*. Reliable experiments, including the use of mutants lacking v-onc genes and transfection assays using cDNA of v-onc genes, have unambiguously demonstrated that these genes are both necessary and sufficient for the transforming ability of such viruses. In addition, use of temperature-sensitive mutants has shown that the expression of protein(s) encoded by the v-onc gene(s) is essential for the expression of the neoplastic phenotype. Furthermore, several systems of regulation of gene expression in transgenic mice have allowed controlled models of neoplasia initiated by numerous oncogenes to be developed in a variety of tissues [15,81]. Retroviruses that carry oncogenes are not a significant cause of naturally-occurring tumors. However, most researchers, stimulated mainly by the discovery in normal cells of protooncogenes homologous to viral oncogenes, have assumed that in all cancers, independently of their etiology and the duration of the preneoplastic process, the critical step driving a normal cell into a neoplastic one must be similar to that carried out by these retroviruses on their target cells [81]. If this were absolutely true, the hypothesis advanced in this paper would again have to be rejected, because that critical step would be a single intracellular event independent of the environment in which the affected cell resides. However, the final word may not have been said yet.

In effect, although signals from v-onc genes have a dominant role in transformation, changes in cellular genes are also required for transformation to occur. This contribution is highlighted by the fact that some v-onc genes fail to transform certain kinds of primary cell cultures but can transform established cell lines derived from them. Similarly, some cellular lineages can be both infected and transformed, while others can be infected but not transformed, by a particular retrovirus carrying a v-onc gene [81]. Furthermore, transgenic animals are usually susceptible to spontaneous tumors involving the tissue (or tissues) in which the transgenic oncogene is expressed. However, in most cases, only a fraction of the animals develop tumors from only a small subset of cells in the infected tissue, and a long latent period is required, indicating that expression of the transgenic oncogene is not sufficient for tumor development. Similar conclusions can be drawn from studies in which a tumor suppressor gene has been selectively disrupted alone or in association with the constitutive expression of a transgenic oncogene [81-83].

A clue to understanding the transforming effect of retroviruses carrying oncogenes to their target cells might be the existence of a common denominator among the different lineages that are both infected and transformed by different retroviruses. In all these lineages, expression of the particular v-onc gene interferes primarily with the normal differentiation of the cells that will be transformed. Conversely, when expression of the v-onc gene fails to arrest the differentiation of the infected cell, no transformation occurs [81]. For example, Abelson murine leukemia virus (Ab-MLV), a virus that normally arrests differentiation of pre-B cells, induces pre-B lymphomas from a small subset of the infected pre-B cells. In contrast, Ab-MLV infects erythroid precursors but does not arrest their differentiation and never induces transformation in this lineage. In fact, expression of the v-abl gene (the v-onc gene of Ab-MLV) can stimulate erythropoeitin-independent differentiation of erythroid cells. Presumably, this reflects the ability of v-Abl protein to mimic signals normally transmitted via the Epo receptor in a situation where the oncoprotein cannot stimulate continued growth [81].

On the basis of the above considerations, we will now advance an interpretation of retroviral carcinogenesis according to the postulates of our hypothesis. Consider a schematic representation of a single hematopoietic normal cell lineage, comprising a stem cell, some undifferentiated mitotically active cells and some differentiated and functional postmitotic cells. The regulation of cell maturation and turnover in a lineage is not completely understood at the molecular level. Nevertheless, the differentiated cells of the lineage somehow control the proliferation of the less differentiated ones [51,84]. For example, when a differentiated cell dies, a restorative signal is generated that induces an undifferentiated cell to divide; one of the resulting cells will differentiate into a functional postmitotic cell while the other will remain undifferentiated, restoring the original function and structure of the lineage.

However, when a retrovirus carrying a v-onc gene infects undifferentiated cells and arrests their differentiation, the normal program of tissue regeneration will be damaged. In effect, although all differentiated functional cells die, promoting a strong regenerative signal, no undifferentiated cell can now differentiate into functional cells, meaning that this lineage would lose its regenerative capacity. Presumably, at early stages of infection, cells that fail to differentiate could only divide three or four times before dying. At that moment, the stem cell would begin to divide to compensate the loss of undifferentiated cells, but these new undifferentiated cells would again be infected with the virus, rendering them unable to differentiate. As a result, a "crisis" would generate a state of variability, and undifferentiated variants not committed to die after a few mitoses would sooner or later emerge. These variants would divide over and over in response to the regenerative signal, thus generating a neoplastic growth. This suggests that the expression of a v-onc gene could be interpreted otherwise than as a single intracellular event that directly drives a normal cell into an autonomous one, as it usually is. Instead, this expression could be a powerful force primarily arresting normal cell differentiation. Only on that basis would a tumor emerge in a subset of those arrested cells. That an impediment to normal cellular differentiation is an essential element in the formation of malignant tumors has recently been suggested by Harris [85].

All the above considerations suggest that carcinogenesis induced by chemicals, radiation and oncogenic viruses, even retroviruses carrying viral oncogenes, considered as the paradigm of the unified genetic theory of cancer, might be reinterpreted according to the postulates of the hypothesis advanced in this paper.

## Plant tumors

It has long been known that the induction of crown gall tumors by *Agrobacterium tumefaciens *in a wide variety of plants depends on the existence of a wound, because inoculating the bacterium into intact plants rarely, if ever, causes tumors [86-88]. However, the precise role of wounding in each step of the tumorigenic process remains unclear.

The conventional interpretation states that the wound is necessary for transformation but not for tumor growth itself. In effect, previous experiments have suggested that phenolic compounds released from the wound trigger both the attachment of *A. tumefaciens *to plant cells and the expression of the *vir *regulon, which is necessary for transferring the oncogenic T-DNA from the bacterium to the cells [86,89]. However, no role in the proliferation of transformed plant cells has been attributed to wounding, since crown gall tumor growth has usually been assumed to depend only on the plant growth hormones produced by the proper transformed cells.

This interpretation contradicts the concept of tumor cells advocated in this paper. However, more recent evidence seems to offer a different picture. *A. tumefaciens *was inoculated in unwounded tobacco seedlings and new molecular technologies were used to demonstrate that *vir *gene induction, T-DNA transfer and plant cell transformation were produced as they are in wounded plants. In contrast to wound sites, the transformed plant cells *could not produce tumors *[88], suggesting that, as long as tissue architecture is not disrupted, negative regulatory signals prevent growth of the transformed cells. On the other hand, such negative regulatory signals would tend to be reduced at wound sites, and proliferation of transformed cells could be initiated in consequence. Since growing galls retard or inhibit the development of normal host tissues [90], transformed cells would have a selective advantage to proliferate, and in consequence the wound would tend to be filled only with transformed cells, which (as opposed to normal wound-healing meristematic cells) display a limited ability to differentiate [86,88]. From that moment, tumor growth could proceed as described in the section "Origin of tumor cells", suggesting that the hypothesis presented in this paper might work even beyond the animal kingdom.

## Anti-cancer treatments

Despite many years of basic and clinical research and trials of promising new therapies, most cancers are resistant to therapy at presentation or become resistant after an initial response [12,91,92]. All current conventional therapies against cancer attempt to kill *all *cancer cells with minimal toxic side effects. A similar aim is pursued by some of the new anti-cancer trials. However, according to our hypothesis, even if all tumor cells were eradicated, the problem might not be solved. In effect, if the organ failure remained, new tumor cells would emerge and the progressive tumor growth would be re-initiated in response to the permanent regenerative signal of the non-restored organ.

A theoretically attractive approach would be to make tumor cells functional, because in that case the organ function would be restored and no regenerative signal would remain to promote new cellular growth. This therapeutic schedule is exemplified by the successful treatment of acute promyelocytic leukemia by retinoic acid-based therapies, resulting in the induction of differentiation of promyelocytes to mature cells [93]. However, in general, attempts to differentiate tumor cells *in vitro *or *in vivo *by using differentiating molecules have proved very difficult [15,52]. On the other hand, full-blown tumor cells become functional or behave as normal cells in the context of strong regenerative fields, stressing that the field in which tumor cells originate, and not only the tumor cells themselves, should be an important objective of our studies [15]. The possibility of manipulating the microenvironment and therefore the growth status of the cell has been demonstrated, for example, with cells that overexpress the hyaluronan receptor [94].

Does this mean that an efficient anti-cancer treatment could be achieved by correcting the organ failure only? Perhaps it depends on the stage of tumor progression. In effect, to correct organ failure means that normal cells can resume their previously lost regenerative capacities and normal organ function and/or structure can be restored. Theoretically, this correction would stop tumor growth because the regenerative signals – which, according to our hypothesis, always guide that growth – would have disappeared. However, that hopeful result could only be achieved if we faced tumor cells at the beginning of tumor growth. Later, when the tumor cells have evolved to become invasive and metastatic, they will have acquired particular features endowing them with the capacity to produce new organ failures. In consequence, to correct the organ failure only would be a transient solution; it would work only until tumor cells damaged the organ again, demanding new tumor growth.

A better putative therapy against full-blown tumor cells might combine the correction of organ failure with an attack against the tumor cells themselves. Perhaps it would not be necessary to eradicate *all *the tumor cells; perhaps it would be enough to lower their number below a critical threshold (tumor dose 50?) by cytostatic rather than cytotoxic therapies, thus avoiding or reducing the occurrence of new organ injuries that would vitiate attempts to correct the organ failure. Below this threshold, cancer cells would presumably be unable to exert any deleterious effect on the organ. In consequence, if the organ failure were corrected, no new failure would be expected and any remaining tumor cells would therefore behave as normal ones.

However, to correct organ failure will demand understanding of the so far elusive nature of the regulatory fields operating in normal organs. Hopefully, the study of wound and wound healing-like phenomena in three-dimensional organotypic cultures [95], which maintain or mimic the natural organ structure, may provide valuable insights into the basic mechanisms of those fields.

## Conclusion

Despite their obvious differences and with few exceptions [14], most theories about cancer proposed during the last century share a common denominator: cancer is believed to be a **biological nonsense **for the organism in which it originates, since cancer cells are believed to be ones that evade the rules controlling normal cell proliferation and differentiation. According to that understanding, cancer cells have usually been considered as cells returning to a more primitive, unicellular, condition of life. Different features of tumor cells such as asocial behavior, reduced need for putative growth factors, ability to grow in overcrowded settings, lack of contact inhibition, etc., have been interpreted by most researchers as confirmation of that primitive way of life.

In contrast, according to the hypothesis advanced in this paper, cancer would have a profound **biological sense**: it would be the ultimate attempt to restore the organ function and structure that have been lost or altered by aging or environmental noxious agents, that is, an attempt to evade the aging and the death of the organ or the organism (if the organ is essential for survival). Therefore, the above-mentioned features of tumor cells could be reinterpreted as progressively acquired adaptations for responding to a permanent regenerative signal in the context of tissue injury, just as several embryonic developmental stages such as morphological differentiation and modeling events could depend on cellular damage and death together with disruption of the topographic field [96]. However, unlike normal structures, cancer would have **no physiological value**, because the usually poorly functional or non-functional nature of its cells would make their reparative task unattainable.

Under special circumstances, however, the attempt of tumors to correct organ failure or to evade death could have been successful. For example, fossil fish of the genus *Pachylebias *that lived 8 million years ago adopted pachyostosis to facilitate immersion in the hyper-saline water of the Mediterranean Sea, "through the development of diffuse hyperostosis that did not differ from a neoplastic form of benign tumor originating from bone tissue" [1]. Similarly, mammals of the Sirenidae group from the Oligocene acquired tumor-like forms "in the axial skeleton to consent browsing on the bottom in shallow waters" [1].

In other cases, tumors appeared as products of inter-specific associations between pairs of organisms. The classic examples are the insect-induced plant gall tumors, which serve a reproductive function for some groups of insects. They represent a re-differentiation and neo-formation of host tissues, characterized by morphological and histological changes elicited by the developing insect that are unique for both the inducing insect and the affected plant organ. In the more highly developed galls, these self-limiting neoplastic growths are almost comparable in the determinate growth of their structures to a leaf or a fruit [87]. These considerations suggest that some neoplasms could have been adopted as a biological strategy to increase the adaptation of some organisms to difficult environmental conditions, allowing the "pathology" to survive for millions of years.

Numerous predictions of the hypothesis advanced in this manuscript might be experimentally testable. For example: (I) the close relationship between strong regenerative ability and absence of tumors throughout the animal kingdom; (II) the existence of an injury and a decreased regenerative capacity in a whole organ or tissue, or in a part of that organ or tissue, before the emergence therein of a primary or secondary metastatic tumor; (III) the existence of danger signals resulting from a retardation of tissue repair, acceleration of cell loss and functional compromise, inducing hereditable changes (genetic or epigenetic) of some kind in aging or injured cells with diminished or exhausted regenerative capacity; (IV) the existence of cellular heterogeneity and a genomic instability phase in an organ or tissue before the acquisition of a frankly malignant phenotype, and in normal tissues surrounding a tumor; (V) the ability of tumor cells, and the inability of surrounding normally aging cells, to respond to local mitogenic or regenerative signals of the tissue in which the tumor has emerged; (VI) the capacity of transplantable tumor cells to injure and to diminish the regenerative ability of the organ or tissue in which they can grow; (VII) the injurious action of carcinogens on cells of organs and tissues as a prerequisite for inducing neoplastic growth, injury meaning not only partial removal of the organ, massive necrosis or extensive degenerative change, but also less evident deleterious effects such as lost or diminished function of the whole organ or part of the organ, apoptosis, cellular senescence, etc.

## Competing interests

The author(s) declare that they have no competing interests.

## Authors' contributions

The two authors contributed equally to this work and they read and approved the final manuscript.
